# Cases in Practice

**Published:** 1875-08

**Authors:** J. J. Erwin


### CASES IN PRACTICE.

BY J. J. ERWIN.

In dental practice there arise many incidents which, were they submitted in a manner that would bring them before the profession, might result in much benefit and satisfaction; whereas, when secrecy is maintained, none are benefited save he who is fortunate enough to have been the discoverer, and he not so much as he would if his discoveries were submitted for the criticism of others,—though not more competent than himself; ideas may be gathered even from our inferiors, which, were they applied in the proper manner and place, would prove to be of great advantage. Whether these remarks may be illustrated in the case supplemented, remains for the reader to determine.

Mrs. J. R., aged about twenty; teeth of frail, glassy, light blue color, called at my office according to engagement, and submitted herself for the filling of the right superior, second molar, which was slightly decayed in central crown. Never having caused her any pain or other inconvenience, we apprehended nothing more than an ordinary case of crown filling, and proceeded with rubber dam, clamps, etc., when, by following the decay into the substance of the tooth, though slight externally, it penetrated to, and revealed the pulp cavity destitute of any substance whatever; not even the trace of a membrane to show there had once been a vital substance there.

The chamber was perfectly formed, dry and emitted no odor or other signs of previous decomposition. As there were no traces of inflammation, we proceeded to fill with gold at once; and to date, the parts have remained normal and manifest no contrary indications for the future.

It is not possible that the tooth was formed without the necessary vital support, yet what became of it without some indications of inflammation or even suppuration, the evidence of the case does not satisfactorily show.

Also, Mrs. B., aged about twenty-two, temperament similar, (and from the effects of a prolonged attack of *facial neuraligia*, had grown exceedingly nervous,) presented herself June 12th, for the purpose of having her teeth extracted preparatory to the insertion of a full artificial denture. On examination I found but four which were free from decay, the remainder of which presented a mass of corruption, with gums of a purple hue and exuding puss which emitted an odor indicative of a prolonged state of disease.

I proceeded at once to rid the mouth of this corruption and in so doing revealed a right inferior cuspid bifurcated one half the length of the root. The specimen I donate to your college museum, as a rarity not often met with. The neuralgia which heretofore has baffled all medical treatment I think had its origin in these teeth and anticipate a speedy recovery.

---

BOSTON, June 29, 1875.

*To the Editor of the Dental Register:*

*Dear Sir.*—At a Union Convention of the Mass. Dental Society and the Merrimack Valley Dental Association held at Lawrence, June 15, 1875, the enclosed resolution was passed. In compliance with that vote I forward it to you with the request to publish in your Journal. Yours Truly,

Geo. F. Grant.

Recording Secretary Mass. Dental Society, 224 Cambridge Street.

*Resolved*, That we, the members of the Massachusetts and Merrimack Valley Dental Societies, in Convention assembled, recommend to all dental practitioners, the discontinuance of the use of Rubber as a base for artificial teeth and the adoption in place thereof of Celluloid, believing this course would not only be for the best interest of ourselves, but for our patients whom we serve.

Voted.—On motion of Dr. McDougal, that a copy of the above resolution be sent to all the dental journals of the country with a request to publish.

